# Directionality of causal association between adolescent mental health and attention deficit: an empirical analysis using a hybrid network model

**DOI:** 10.3389/fpsyg.2025.1658007

**Published:** 2025-10-16

**Authors:** Miaomiao Li, Jing Lv, Shaoxiong Li, Congcong Liu, Ziyan Wang, Zheng Liu, Huan Liu, Xuan Liu, Yuru Du, Youdong Li

**Affiliations:** ^1^Clinical Mental Health Department, The First Hospital of Hebei Medical University, Hebei, China; ^2^College of Education, Hebei Normal University, Shijiazhuang, China

**Keywords:** adolescent mental health, attention deficit, Bayesian network, psychological network, anxiety, paranoia, interpersonal sensitivity

## Abstract

**Objective:**

By constructing undirected and Bayesian network models, this study overcomes the limitations of traditional correlation analyses, revealing the underlying causal relationships and operational mechanisms between adolescent mental health and attention deficits.

**Methods:**

A total of 19,157 valid responses (effective response rate: 96.92%) were collected from adolescents aged 11–20 (<0.1% aged 20) at secondary schools in a Hebei Province region via the Wenjuanxing platform. Mental health (10 dimensions) and attention problems (3 dimensions) were assessed using the *Mental Health Scale for Secondary School Students* in China (MSSMHS; 60 items, *α* = 0.976) and the *Swanson, Nolan, and Pelham Rating Scale-IV* (SNAP-IV-26; 26 items, α = 0.942). In R Studio, an undirected network was constructed using the EBICglasso algorithm (with regularization and 1,000 bootstrap tests), and centrality analysis identified core variables. A directed acyclic graph (DAG) was generated via Bayesian network analysis (hill-climbing algorithm) with 50 random restarts, 100 perturbations, and 100 bootstrap validations (edge stability threshold: 0.85; edges retained only if present in ≥85% of subsamples), elucidating causal pathways between mental health and attention deficits.

**Results:**

Undirected network analysis revealed the strongest associations between depression and anxiety, hyperactivity/impulsivity and oppositional defiant behavior, and paranoia and interpersonal sensitivity. Centrality metrics showed anxiety with the highest strength centrality, paranoia with the highest closeness centrality and betweenness centrality. In the Bayesian DAG, interpersonal sensitivity, anxiety, and paranoia occupied the top hierarchical level, connecting to intermediate nodes (e.g., hostility, emotional instability, sense of academic pressure) and ultimately to terminal nodes (attention deficit, oppositional defiant behavior, psychological imbalance, hyperactivity/impulsivity).

**Conclusion:**

This study demonstrates that adolescent mental health influences attention deficits through multiple distinct causal pathways: ① interpersonal sensitivity, anxiety, emotional instability, and hostility lead to inattention; ② anxiety, paranoia, depression, and hostility contribute to hyperactivity/impulsivity; ③ interpersonal sensitivity, hostility, depression, emotional instability, and sense of academic pressure result in oppositional defiant behaviors. These findings identify precise intervention targets for distinct dimensions of attention deficits in adolescents and provide mechanistic empirical evidence for understanding the multidimensional causal architecture through which psychological symptoms impact behavioral outcomes.

## 1 Introduction

Adolescence represents a critical phase for cognitive and emotional development, during which mental health risks are garnering increased concern (Crone and Dahl, 2012; Miller and Prinstein, 2019). Studies indicate rising prevalence rates of depression, anxiety, and related issues among Chinese adolescents, with a notable trend toward earlier onset (Oliveira et al., 2022; Yu, 2022). Despite sustained national efforts to strengthen mental health initiatives (National Health Commission of the People’s Republic of China, 2019a; National Health Commission of the People’s Republic of China, 2019b; Ministry of Education of the People’s Republic of China, 2023), persisting societal challenges—particularly social isolation and educational disruptions during the COVID-19 pandemic—have been empirically shown to exacerbate psychological risks (Rothenberg et al., 2023; Meherali et al., 2021). Ecological systems theory posits that such issues originate from dynamic interactions among individual traits, familial dynamics, academic pressures, and sociocultural factors (World Health Organization, 2025; Yu et al., 2018). Within this context, the relationship between adolescent mental health and specific cognitive difficulties—namely attention problems characterized by challenges in sustaining focus and inhibiting distractions—demands rigorous investigation. It should be noted that these attention problems behaviorally overlap significantly with the predominantly inattentive subtype of ADHD (ADHD-I). Executive function theory provides a mechanistic explanation: impairments in core abilities (e.g., inhibitory control) may directly underlie difficulties in maintaining attention (Wang and Zhou, 2007), while emotional regulation disorders such as anxiety and interpersonal sensitivity could amplify this process (Biederman, 2005). Simultaneously, epidemiological data corroborate the significance of this association: The overall prevalence of Attention-Deficit/Hyperactivity Disorder (ADHD) among Chinese children and adolescents stands at 3.6% (Dong et al., 2025). Although lower than the global prevalence rate for the same age group (5.6%; Salari et al., 2023), the absolute number of affected individuals remains substantial due to China’s large population base. Notably, the predominantly inattentive presentation (ADHD-I) constitutes a prominent proportion of cases. These attention problems demonstrate significant associations with school maladjustment (e.g., school refusal behavior; Du et al., 2024; Erskine et al., 2016), underscoring the public health imperative to elucidate mental health–attention mechanisms.

Nevertheless, current research faces challenges in delineating the link between mental health and attention problems. While cross-sectional analyses can reveal statistical associations, they cannot systematically verify specific causal pathways through which mental health symptoms influence attention problems (Santos et al., 2023; Chacko et al., 2024). The emergence of network methodologies offers new analytical avenues (Cramer et al., 2010), modeling psychological constructs as nodes and their interrelations as edges to map direct and indirect connections (Borsboom, 2008). This approach clarifies interactions among observable variables while mitigating latent confounds. Undirected networks (e.g., Gaussian Graphical Models) remain valuable for exploratory variable screening and cluster identification (Briganti et al., 2023) but cannot infer causality due to edge symmetry. To address this, we implement a sequential “exploration-verification” strategy: initial undirected network analysis identifies core variables and structural patterns, followed by Bayesian Networks (BNs; Newman, 2003)—directed probabilistic graphical models—which estimate causal directions and effect magnitudes under stricter assumptions (Kitson et al., 2023). While BNs facilitate causal inference, we acknowledge inherent limitations with observational data. Collectively, these methods map latent causal pathways among psychological variables.

This study integrates hybrid network modeling to investigate how mental health symptoms influence attention problems in adolescents. By identifying key associations through an undirected network and subsequently probing directional pathways via BN analysis, we aim to advance psychobehavioral interaction theories. Specifically, we focus on mental health-to–attention problem pathways to inform targeted early interventions and mental health promotion strategies.

## 2 Materials and methods

### 2.1 Participants

This study utilized a convenience sampling approach, recruiting middle school students from Hebei Province, China. Data were collected via *Wenjuanxing*, an online survey platform. A total of 19,766 responses were initially obtained. After excluding responses with missing values or repetitive patterns (e.g., identical answers across items), 19,157 valid datasets were retained, yielding an effective response rate of 96.92%. Among the valid responses, 9,645 (50.3%) were boys and 9,512 (49.7%) were girls; 71.8% were junior-high and 28.2% senior-high students. Participants were aged 11–20 years (M = 14.7, SD = 1.5), with only five individuals (≈0.03%) being 20 years old. Per the WHO definition (10–19 years), the sample technically includes five participants beyond the adolescent range; for brevity, we hereafter refer to the entire cohort as “adolescents.”

### 2.2 Methods

#### 2.2.1 Chinese middle school student mental health scale

The MSSMHS was developed by Professor Wang Jisheng, a renowned psychologist at the Institute of Psychology, Chinese Academy of Sciences (Wang et al., 1997). Tailored to the cultural and behavioral characteristics of Chinese adolescents, this symptom-oriented psychological diagnostic tool is designed to screen for psychological issues in middle school students. The scale comprises 60 items across 10 dimensions: Compulsive symptoms, Paranoia, Hostility, Interpersonal sensitivity, Depression, Anxiety, Sense of academic pressure, Poor adaptation, Emotional instability, Psychological imbalance. Previous studies have established strong reliability and validity for the scale (Wang et al., 1997). In the current study, both the full scale and subscales demonstrated robust psychometric properties, with detailed reliability and validity indices presented in Table 1.

**Table 1:** Reliability and validity of the Chinese middle school student mental health scale (MSSMHS) and its subscales.

Scale / Subscale		Reliability	Validity		Number of items
		(Cronbach’s *α*)	KMO	Bartlett’s test of sphericity (*p*-value)	
Full scale		0.976	0.987	< 0.001	60
Subscales	Compulsive symptoms	0.732	0.796	< 0.001	6
	Paranoia	0.843	0.877	< 0.001	6
	Hostility	0.855	0.862	< 0.001	6
	Interpersonal sensitivity	0.816	0.858	< 0.001	6
	Depression	0.871	0.901	< 0.001	6
	Anxiety	0.907	0.914	< 0.001	6
	Sense of academic pressure	0.881	0.864	< 0.001	6
	Poor adaptation	0.783	0.847	< 0.001	6
	Emotional instability	0.830	0.849	< 0.001	6
	Psychological imbalance	0.764	0.841	< 0.001	6

#### 2.2.2 Swanson, Nolan, and Pelham rating scale version IV (SNAP-IV-26)

The SNAP-IV-26 is a widely used assessment tool based on the *Diagnostic and Statistical Manual of Mental Disorders* (DSM-IV) criteria, designed to evaluate adolescent attention deficit, hyperactivity/impulsivity, and oppositional defiant behavior (Hall et al., 2020). This 26-item scale comprises three subscales corresponding to the aforementioned dimensions. Prior research supports its strong psychometric properties, making it a reliable instrument for mental health and attention-related studies (Zhou et al., 2013). In the current study, the full scale and all subscales demonstrated robust reliability and validity, with detailed indices presented in Table 2.

**Table 2:** Reliability and validity of the Swanson, Nolan, and Pelham rating scale (SNAP-IV-26) and its subscales.

Scale / Subscale		Reliability	Validity		Number of items
		(Cronbach’s α)	KMO	Bartlett’s test of sphericity (*p*-value)	
Full scale		0.942	0.968	< 0.001	26
Subscales	Attention deficit	0.893	0.931	< 0.001	9
	Hyperactivity/impulsivity	0.846	0.908	< 0.001	9
	Oppositional defiant	0.883	0.897	< 0.001	8

### 2.3 Data analysis

The analyses were conducted in *RStudio* (R version 4.4.2) using two complementary network modeling approaches.

#### 2.3.1 Undirected network estimation

*Gaussian Graphical Models* (GGMs) have become essential tools for exploratory psychological network analysis, with their validity and widespread application demonstrated in multiple studies (Chacko et al., 2024; Cramer et al., 2010; Borsboom, 2008). Within this framework, we estimated an undirected network using the *Extended Bayesian Information Criterion with Graphical Lasso* (EBICglasso) model to identify partial correlations between variables. The EBICglasso employs *ℓ₁*-penalized regularization to reduce false-positive connections while maintaining model parsimony. Network visualization was implemented using the qgraph package.

Node centrality—a measure of a node’s connectivity and influence within a network—was assessed using three key metrics: strength centrality, which extends degree centrality by incorporating both the number and weight of a node’s connections to evaluate its overall influence; betweenness centrality, reflecting a node’s role as an intermediary in the shortest paths between other nodes and indicating its control over information flow; and closeness centrality, calculated as the inverse of the average shortest path length to all other nodes, which captures a node’s efficiency in network-wide communication (Freeman, 1979; Sadhu et al., 2024). These indices were computed using the qgraph package in R to quantify the relative importance of each node. To ensure robustness, network stability and the reliability of centrality estimates were evaluated through 1,000 bootstrap iterations via the bootnet package, generating confidence intervals for edge weights and centrality metrics (Epskamp et al., 2018). This approach mitigates sampling variability and enhances confidence in the inferred network structure.

#### 2.3.2 Directed network (Bayesian network) estimation

Bayesian Networks have demonstrated preliminary validity in psychological causal research, hence this study adopted this approach (Briganti et al., 2024; Zhang et al., 2025). For the directed network, we implemented the *Hill-Climbing* algorithm through the hc function in the bnlearn package, generating a directed acyclic graph (DAG) to represent causal dependencies (Adhitama and Saputro, 2022). The HC algorithm—a heuristic search method commonly employed for BN estimation—starts from a randomly initialized network structure and iteratively optimizes it by adding, removing, or reversing edges to improve model fit (Gámez et al., 2011). To reduce the risk of converging to local optima, 50 random restarts were implemented, enabling the algorithm to explore multiple initial configurations. 100 perturbations per iteration were introduced to diversify the search process, enhancing the likelihood of identifying the global optimal structure. Network stability was rigorously evaluated through 100 bootstrap replications, and edges consistently appearing in ≥85% of bootstrapped networks were retained to ensure reliability (Heckerman et al., 1995). This comprehensive approach balances computational efficiency with robustness, ensuring both accurate causal inference and reproducibility.

## 3 Results

### 3.1 Structural characteristics and node associations in the undirected network (EBICglasso network, EN)

**Figure 1:**
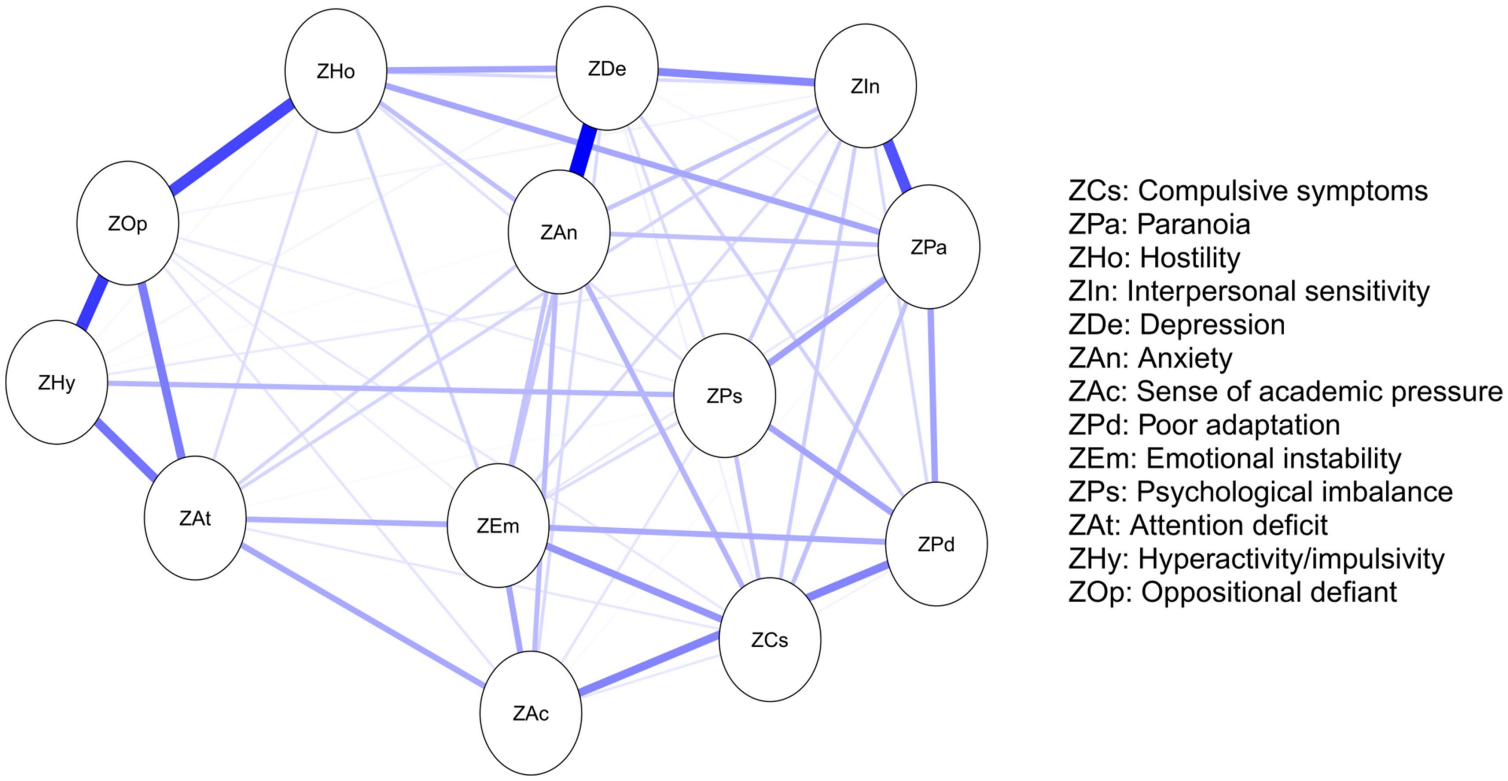
Undirected EBICglasso network model illustrating connectivity between nodes. Each dimension is represented as a circular node in the network, and edges between nodes indicate conditional dependencies. The thickness of edges reflects the strength of associations between symptoms. Blue and red edges denote positive and negative correlations, respectively.

In the undirected network model (Figure 1), thicker edges—indicating stronger connections—were observed between depression and anxiety, hyperactivity/impulsivity and oppositional defiant, oppositional defiant and hostility, as well as paranoia and interpersonal sensitivity. Analysis of the weighted adjacency matrix (Table 3) further confirmed these associations, with higher edge weights for these node pairs compared to others, underscoring their robust connectivity. These findings suggest that depression, anxiety, hyperactivity/impulsivity, oppositional defiant, hostility, paranoia, and interpersonal sensitivity serve as pivotal nodes in information transmission and functional integration within the current network model.

**Table 3:** EBICglasso weighted adjacency matrix.

Node	ZCs/ZCosy	ZPa	ZHo	ZIn/ZInse	ZDe	ZAn	ZAc/ZAcpr	ZPd/ZPoad	ZEm/ZEmim	ZPs/ZPsim	ZAt/ZAtde	ZHy/ZHylm	ZOp/ZOpde
ZCs/ZCosy	0.00												
ZPa	0.11	0.00											
ZHo	0.00	0.16	0.00										
ZIn/ZInse	0.09	**0.31**	0.07	0.00									
ZDe	0.03	0.01	0.16	0.21	0.00								
ZAn	0.13	0.11	0.11	0.10	**0.45**	0.00							
ZAc/ZAcpr	0.04	−0.01	0.00	0.00	0.07	0.12	0.00						
ZPd/ZPoad	0.02	0.16	0.00	0.07	0.08	0.00	0.22	0.00					
ZEm/ZEmim	0.19	0.04	0.08	0.06	0.10	0.11	0.16	0.15	0.00				
ZPs/ZPsim	0.10	0.17	0.05	0.09	−0.06	0.00	0.05	0.16	0.06	0.00			
ZAt/ZAtde	0.04	0.00	−0.06	0.07	0.00	0.07	0.15	0.02	0.14	−0.01	0.00		
ZHy/ZHylm	0.00	0.04	0.01	0.00	−0.02	−0.01	0.00	0.00	0.00	0.13	0.25	0.00	
ZOp/ZOpde	−0.04	0.00	**0.33**	0.02	0.00	0.00	0.05	0.00	0.03	0.04	0.23	**0.35**	0.00

Bold values indicate edge weights that are higher than the remaining connections, reflecting stronger functional connectivity and greater information-transfer capacity between the corresponding nodes.

**Figure 2:**
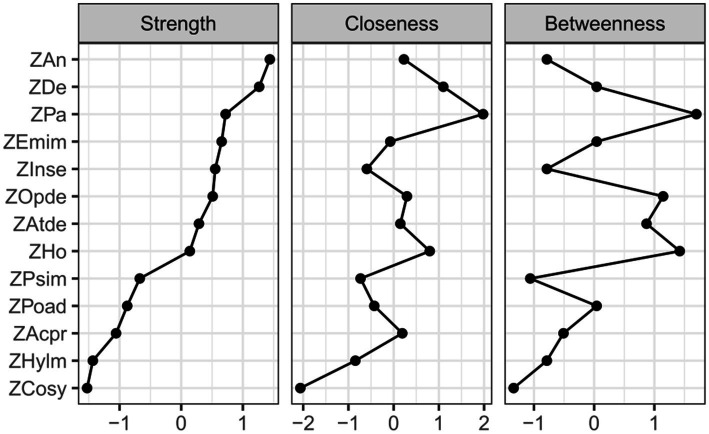
Analysis of standardized centrality metrics (strength, closeness, and betweenness centrality). The y-axis represents the 13 dimensions from the MSSMHS and SNAP-IV-26, while the x-axis denotes the standardized values of centrality metrics.

To better understand the roles of these nodes within the network, this study calculated standardized centrality metrics of strength, closeness, and betweenness centrality (Figure 2). Centrality analysis of the adolescent mental health network revealed that anxiety, depression, and paranoia exhibited significantly higher strength centrality than other nodes (*p* < 0.01), indicating these psychological features possess greater connectivity and influence within the network, substantially impacting the system’s overall functionality and stability. Furthermore, paranoia, depression, and hostility showed significantly higher closeness centrality than other nodes (*p* < 0.01), suggesting these nodes have superior information transmission efficiency and can rapidly disseminate information across the network, thereby enhancing the system’s overall responsiveness. In the betweenness centrality analysis, paranoia, hostility, and oppositional defiant were notably elevated (*p* < 0.01), signifying their critical bridging roles in connecting different nodes and subsystems, facilitating information flow and resource integration, which are vital for systemic coordination. Importantly, paranoia and depression frequently co-occur in adolescents. The paranoid psychological characteristics may exacerbate depressive symptoms, and vice versa. This comorbidity could potentially lead to more complex mental health challenges (Xie et al., 2024).

**Figure 3:**
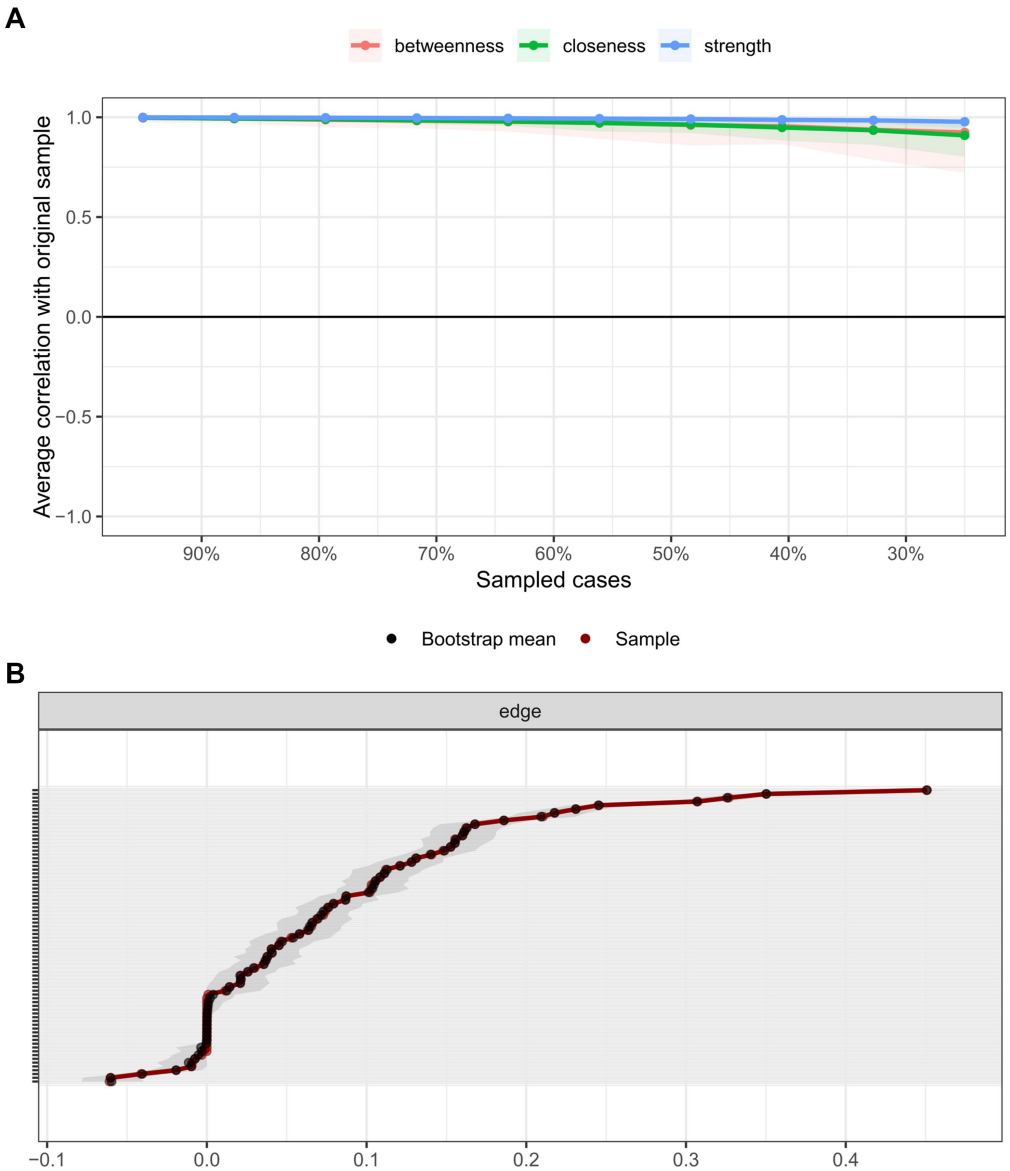
**(A)** Stability assessment of centrality metrics. A graphical summary of the average correlations for centrality metrics across subsampling proportions, using bootstrap estimates of centrality from the original network. Horizontal lines represent the mean correlation, while vertical bars delineate the 2.5th to 97.5th percentile intervals. The plot demonstrates that even at a 30% subsampling proportion, the mean correlations for all three centrality metrics (strength, closeness, and betweenness) remain exceptionally high (approaching 1.0). These bootstrap results serve as strong indicators of the reliability of centrality indices. **(B)** Edge weight estimation accuracy summary of differences between original edge weights and bootstrapped estimates. Black lines denote the mean edge strength across bootstrap samples, red lines represent original edge weights from the full sample, and gray shaded areas indicate the 95% confidence intervals for bootstrapped edge strengths. Each horizontal line corresponds to a network edge, ordered from highest to lowest edge weight. Notably, edge strength estimates remain stable for the majority of edges, except for those with extreme weights (e.g., very high or low values). This stability confirms the robustness of edge weight estimation in the network model.

To evaluate the stability of the network structure, 1,000 bootstrap samples were generated to assess the consistency of centrality metrics (Figure 3A) and the accuracy of edge weight estimates (Figure 3B). Figure 3A displays the average correlations between strength, closeness, and betweenness centrality indices across varying subsampling proportions (30–100% of the original sample). The results indicated that even at a reduced subsampling proportion of 30%, correlations for all three centrality metrics remained >0.95, demonstrating exceptional stability. This suggests that centrality indices reliably reflect the network structure despite sample size reductions. Figure 3B illustrates the distribution of edge weight estimates across bootstrap samples. Red dots represent original edge weights, while black dots denote bootstrapped estimates, with gray shaded regions indicating uncertainty intervals. Most bootstrapped edge estimates closely aligned with original values, confirming high estimation accuracy. Notably, uncertainty intervals narrowed progressively for edges with larger weights, further validating the stability of high-weight connections. These findings collectively emphasize the robustness of both centrality metrics and edge weight estimations across varying sample sizes, providing a reliable foundation for interpreting the network’s structural and functional properties.

### 3.2 Causal inference in the Bayesian network

In the directed network model, the relationship between adolescent mental health and attention deficits manifests through multi-level causal pathways. Core nodes—interpersonal sensitivity, anxiety, and paranoia—exert direct causal effects on intermediary nodes (e.g., hostility, emotional instability, sense of academic pressure, poor adaptation, compulsive symptoms), which subsequently transmit influences to behavioral outcome nodes (attention deficit, oppositional defiant, psychological imbalance, hyperactivity/impulsivity). This hierarchical causal cascade underscores the dynamic progression of mental health challenges, wherein upstream psychological traits modulate downstream behavioral manifestations through sequential intermediation.

**Figure 4:**
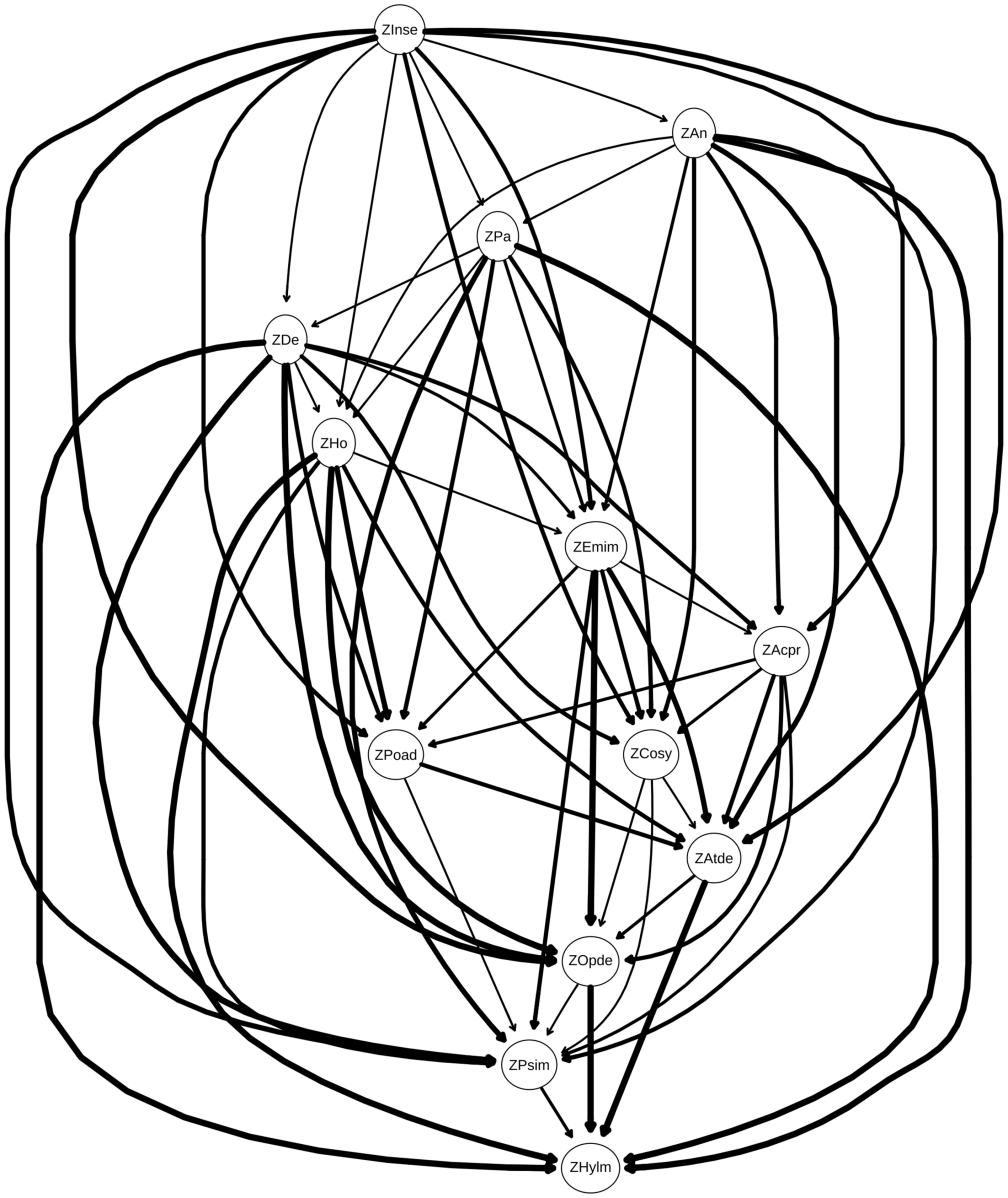
Directed acyclic graph (DAG) illustrating causal relationships between dimensions. Each dimension is represented as a node, with edge thickness corresponding to the magnitude of directional influence between nodes (thicker edges denote stronger effect sizes). Edges are sorted from strongest to weakest based on their weights, as detailed in Table 4. This visualization highlights prioritized causal pathways for targeted intervention while maintaining methodological transparency through supplementary tabular data.

This study employed the *Hill-Climbing* (HC) algorithm to estimate the *Bayesian network* (BN), constructing a *Directed Acyclic Graph* (DAG) as illustrated in Figure 4. Through iterative optimization, the network retained edges with statistical significance and the strongest probabilistic dependencies observed in the data. In the resulting DAG (Figure 4), core nodes at the uppermost layer—interpersonal sensitivity, anxiety, and paranoia—exert causal effects on downstream nodes through prominent pathways, underscoring their pivotal roles in the network. The thickness of edges reflects both the probability of directional causality (i.e., the likelihood of edge direction across bootstrap samples) and the strength of associations, with thicker edges indicating stronger causal linkages and more stable directionality. Nodes at the bottom layer—attention deficit, oppositional defiance, psychological imbalance, and hyperactivity/impulsivity—exhibit significant connections with multiple upstream nodes, suggesting they serve as convergent outcomes of diverse psychological symptoms. These nodes integrate influences from multiple causal pathways, reflecting the complexity of mental health comorbidities. The hierarchical structure of the DAG implies that interventions targeting upstream core nodes (e.g., mitigating interpersonal sensitivity or anxiety) could disrupt cascading effects, potentially alleviating downstream behavioral issues such as attention deficits. By identifying and addressing these pivotal nodes, stakeholders may develop multi-level strategies to enhance adolescent mental health and reduce the burden of attention-related challenges.

To assess the stability of the derived DAG, 100 bootstrap simulations were conducted (Scutari and Nagarajan, 2013). Edges with edge weights > 0.85 and directional probabilities > 0.5 were retained, and their average values were computed to derive the final network structure. This process enhanced network sensitivity by preferentially selecting edges that appeared consistently across bootstrap samples, while improving specificity through a stringent inclusion threshold (*edges present in ≥85% of bootstrapped networks*). Consequently, the averaged network exhibited structural similarity to the original DAG but included additional edges, reducing sparsity and enhancing robustness against sampling variability.

Additionally, edge weights were analyzed to quantify directional certainty and association strength. Edge weights, determined by their directional probabilities (ranging from 0 to 1), ensured non-negativity while reflecting the reliability and significance of causal directions within the data. Edges with directional probabilities > 0.4 and association strengths exceeding the optimal significance threshold were retained, ensuring statistical robustness and directional consistency in the final network. Table 4 lists these edge weights, sorted by association strength, confirming the importance of stability-screened edges in the network. Notably, certain edges in the DAG (Figure 4) exhibit weights > 0.4 but are not visually depicted. For instance, the edge from anxiety to depression has a weight of 0.49, indicating a moderate yet meaningful association. To provide comprehensive insights, Table 4 enumerates all edges with their corresponding weights, elucidating the strength of inter-node relationships. This supplementary data is critical for interpreting network architecture, particularly when reconciling high-weight edges absent from the graphical representation. By synthesizing information from both Figure 4 and Table 4, researchers can unravel the complex interplay among psychological symptoms, advancing mechanistic understanding and informing targeted interventions.

**Table 4:** Edge weights in the Bayesian network (BN).

Edge	From	To	Weight		From	To	Weight		From	To	Weight
1	ZAn	ZHy/ZHylm	1.00	26	ZEm/ZEmim	ZPs/ZPsim	0.81	51	ZEm/ZEmim	ZAc/ZAcpr	0.60
2	ZPa	ZHy/ZHylm	1.00	27	ZHo	ZPs/ZPsim	0.81	52	ZCs/ZCosy	ZAt/ZAtde	0.60
3	ZDe	ZHy/ZHylm	1.00	28	ZPa	ZCs/ZCosy	0.79	53	ZHo	ZEm/ZEmim	0.59
4	ZHo	ZHy/ZHylm	1.00	29	ZDe	ZAc/ZAcpr	0.77	54	ZIn/ZInse	ZPa	0.57
5	ZAt/ZAtde	ZHy/ZHylm	1.00	30	ZAc/ZAcpr	ZAt/ZAtde	0.77	55	ZCs/ZCosy	ZOp/ZOpde	0.55
6	ZIn/ZInse	ZOp/ZOpde	0.98	31	ZIn/ZInse	ZAc/ZAcpr	0.76	56	ZPa	ZHo	0.55
7	ZHo	ZOp/ZOpde	0.98	32	ZPd/ZPoad	ZAt/ZAtde	0.75	57	ZIn/ZInse	ZAn	0.55
8	ZDe	ZOp/ZOpde	0.97	33	ZAn	ZAc/ZAcpr	0.75	58	ZIn/ZInse	ZDe	0.53
9	ZDe	ZPs/ZPsim	0.96	34	ZEm/ZEmim	ZCs/ZCosy	0.75	59	ZAn	ZPa	0.53
10	ZEm/ZEmim	ZOp/ZOpde	0.95	35	ZDe	ZCs/ZCosy	0.74	60	ZDe	ZAn	0.52
11	ZOp/ZOpde	ZHy/ZHylm	0.95	36	ZIn/ZInse	ZEm/ZEmim	0.74	61	ZPa	ZDe	0.51
12	ZIn/ZInse	ZAt/ZAtde	0.94	37	ZDe	ZEm/ZEmim	0.72	62	ZOp/ZOpde	ZPs/ZPsim	0.51
13	ZAn	ZAt/ZAtde	0.94	38	ZPs/ZPsim	ZHy/ZHylm	0.72	63	ZPs/ZPsim	ZOp/ZOpde	0.49
14	ZEm/ZEmim	ZAt/ZAtde	0.93	39	ZEm/ZEmim	ZPd/ZPoad	0.71	64	ZDe	ZPa	0.49
15	ZHo	ZPd/ZPoad	0.92	40	ZAn	ZEm/ZEmim	0.70	65	ZAn	ZDe	0.49
16	ZPa	ZPs/ZPsim	0.91	41	ZAc/ZAcpr	ZPd/ZPoad	0.70	66	ZPa	ZAn	0.48
17	ZIn/ZInse	ZPs/ZPsim	0.91	42	ZAc/ZAcpr	ZCs/ZCosy	0.70	67	ZDe	ZIn/ZInse	0.47
18	ZHo	ZAt/ZAtde	0.89	43	ZAc/ZAcpr	ZPs/ZPsim	0.69	68	ZAn	ZIn/ZInse	0.46
19	ZAn	ZPs/ZPsim	0.89	44	ZPa	ZEm/ZEmim	0.67	69	ZHo	ZPa	0.45
20	ZAc/ZAcpr	ZOp/ZOpde	0.87	45	ZAt/ZAtde	ZOp/ZOpde	0.67	70	ZOp/ZOpde	ZCs/ZCosy	0.45
21	ZIn/ZInse	ZPd/ZPoad	0.84	46	ZIn/ZInse	ZHo	0.65	71	ZPa	ZIn/ZInse	0.43
22	ZIn/ZInse	ZCs/ZCosy	0.84	47	ZPd/ZPoad	ZPs/ZPsim	0.63	72	ZEm/ZEmim	ZHo	0.41
23	ZDe	ZPd/ZPoad	0.83	48	ZAn	ZHo	0.62	73	ZAt/ZAtde	ZCs/ZCosy	0.40
24	ZAn	ZCs/ZCosy	0.83	49	ZDe	ZHo	0.62				
25	ZPa	ZPd/ZPoad	0.81	50	ZCs/ZCosy	ZPs/ZPsim	0.61				

### 3.3 Cross-dimensional bridging mechanisms of the bridging network

**Figure 5:**
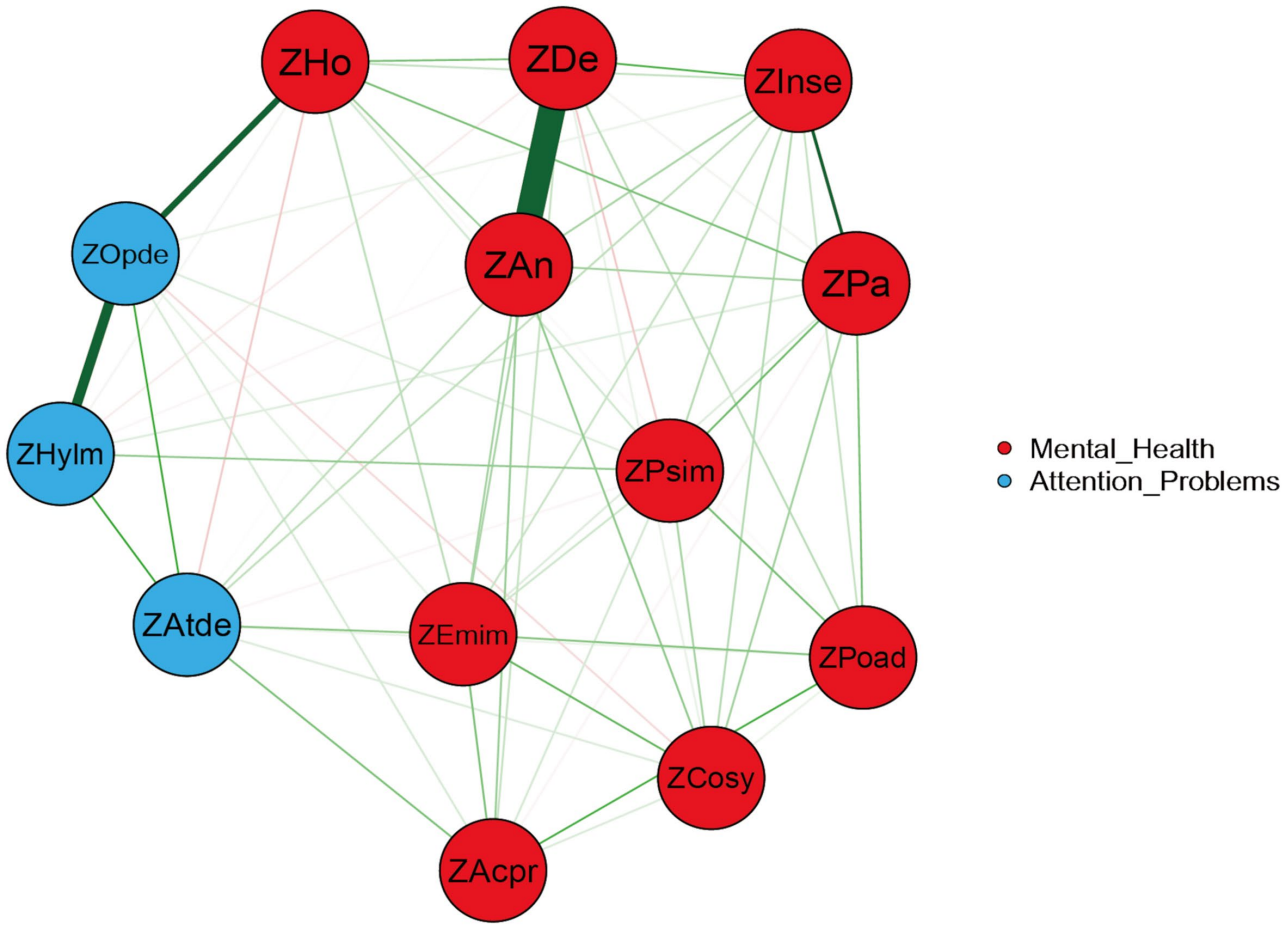
Bridging network structure between mental health and attention problems. Red nodes represent variables within the mental health dimension. Blue nodes represent variables within the attention problems dimension. Edge thickness indicates the strength of association between nodes.

**Figure 6:**
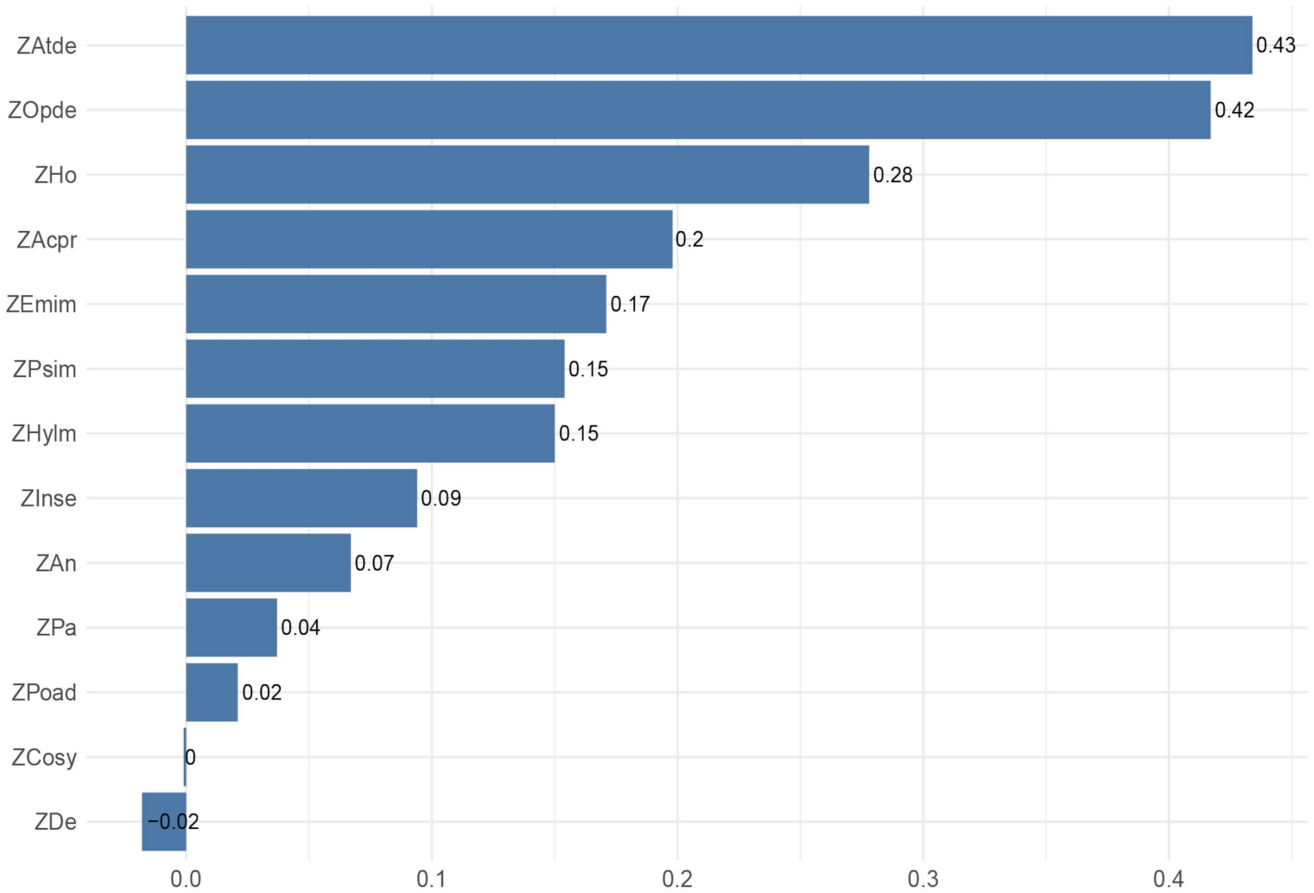
Distribution of node Bridging Expected Influence (BEI) values. Nodes are ordered by descending BEI value. Bar height represents the magnitude of the BEI value.

This study employed bridging centrality analysis to identify key nodes bridging the association between mental health problems and attention-related issues (Yang et al., 2024). As presented in Table 5, the bridging expected influence (BEI) values ranked highest for Attention deficit (0.434), Oppositional defiant (0.417), and Hostility (0.278). Similarly, bridging strength was highest for Attention deficit (0.573), Oppositional defiant (0.500), and Hostility (0.398). The 95% confidence intervals (CI), calculated via 100 bootstrap samples, indicated that Emotional instability exhibited the narrowest CI (−0.032, 0), suggesting high stability in its bridging effect. In contrast, Depression had the widest CI (0.389, 0.445), indicating greater variability in its result. The bridging network structure between mental health and attention problems (Figure 5) revealed the thickest edge connecting Anxiety and Depression within the mental health dimension. Relatively thicker cross-dimension edges were observed between Poor adaptation and Hyperactivity/impulsivity, and between Oppositional defiant and Hostility. The distribution of node bridging expected influence (Figure 6) demonstrated that Attention deficit (0.43) and Oppositional defiant (0.42) possessed the highest BEI values, indicating the strongest potential for cross-dimension influence. Hostility (0.28) ranked third. The negative BEI value for Depression (−0.02) signifies its minimal impact across dimensions.

**Table 5:** Node bridging characteristics and stability analysis.

Node	BEI	Bridge_Strength	95% confidence interval (CI)
ZAt/ZAtde	0.434	0.573	(−0.024, 0.013)
ZOp/ZOpde	0.417	0.5	(0.019, 0.054)
ZHo	0.278	0.398	(0.256, 0.302)
ZAc/ZAcpr	0.198	0.198	(0.078, 0.114)
ZEm/ZEmim	0.171	0.171	(−0.032, 0)
ZPs/ZPsim	0.154	0.173	(0.046, 0.084)
ZHy/ZHylm	0.15	0.203	(0.177, 0.218)
ZIn/ZInse	0.094	0.094	(0.004, 0.038)
ZAn	0.067	0.082	(0.148, 0.185)
ZPa	0.037	0.037	(0.136, 0.18)
ZPd/ZPoad	0.021	0.021	(0.408, 0.455)
ZCs/ZCosy	−0.001	0.082	(0.124, 0.174)
ZDe	−0.018	0.02	(0.389, 0.445)

BEI, Bridging Expected Influence, quantifying the extent to which a node acts as a hub in cross-dimension bridging. Values range ∈ (−1, 1), with positive values indicating an outward bridging function. The 95% CI was calculated based on 100 bootstrap samples.

## 4 Discussion

This study investigated the intricate relationship between adolescent mental health and attention deficits through the construction of undirected networks and Bayesian networks (BN). Utilizing the *Chinese Middle School Student Mental Health Scale* (MSSMHS) and the *Swanson, Nolan, and Pelham Rating Scale* (SNAP-IV-26), we identified pivotal roles of core nodes—such as anxiety, depression, paranoia, and interpersonal sensitivity—in network connectivity and their underlying causal mechanisms. These nodes exhibited significant centrality (strength, closeness, betweenness), underscoring their critical roles in information transmission and functional integration. Notably, anxiety and paranoia, positioned as top-tier nodes in the BN, exerted substantial downstream influence, suggesting their centrality in the onset and perpetuation of psychological symptoms. The causal pathways inferred from the BN revealed dynamic mechanisms through which core nodes propagate effects. For instance, interpersonal sensitivity may amplify anxiety and paranoia, indirectly exacerbating comorbid symptoms, while anxiety could intensify hostility and perceptions of academic pressure, thereby undermining mental health. Similarly, paranoia might directly elevate depressive symptoms and maladaptive behaviors, compounding psychological distress. Additionally, attention deficits emerged as a critical mediator in these pathways: influenced by upstream nodes such as anxiety, depression, and hostility, attention deficits further exacerbated downstream issues like hyperactivity/impulsivity and oppositional defiance, amplifying the complexity of mental health challenges. These findings provide theoretical grounding for future interventions, emphasizing the need to target core nodes (e.g., anxiety, paranoia) to disrupt cascading effects. For example, reducing interpersonal sensitivity could mitigate anxiety and paranoia, while addressing academic pressure might alleviate hostility and emotional instability. Furthermore, interventions for attention deficits should integrate strategies to modify upstream psychological traits (e.g., depressive cognitions, hostile attitudes) rather than focusing solely on behavioral symptoms. By elucidating these hierarchical and reciprocal relationships, our study advances a holistic framework for understanding and addressing adolescent mental health comorbidities.

A comparison of Figures 1, 4 reveals that most connections in the undirected network persist in the DAG. The symptom centrality indices observed in the undirected network exhibit clear correspondences with directional influences in the DAG: nodes central in the undirected network also demonstrate significant causal impacts in the directed model, indicating cross-model stability of key nodes.

In the undirected network, thicker edges—representing stronger associations—were observed between depression and anxiety, hyperactivity/impulsivity and oppositional defiance, oppositional defiance and hostility, and paranoia and interpersonal sensitivity. These robust connections suggest mutually reinforcing interactions between these symptoms or psychological states.

Notably, depression and anxiety share overlapping symptomatology (e.g., insomnia, attention deficits, fatigue), which may act as *bridging mechanisms* that facilitate mutual activation of depressive and anxious symptom clusters (Groen et al., 2020). Anxiety may perpetuate feelings of helplessness and insecurity, exacerbating depressive symptoms, while depression could amplify pessimistic outlooks, further intensifying anxiety. Consequently, therapeutic interventions should adopt a holistic approach that addresses both conditions simultaneously, targeting shared symptoms (e.g., sleep disturbances, cognitive fatigue) to disrupt their reciprocal reinforcement. This bidirectional relationship underscores the importance of transdiagnostic strategies in adolescent mental health care, moving beyond symptom-specific treatments to address underlying cognitive and emotional synergies.

Adolescents exhibiting hyperactivity/impulsivity often display behaviors such as excessive restlessness, difficulty remaining seated, and impulsive actions, while those with oppositional defiance frequently challenge authority, defy rules, and disregard instructions. These behavioral manifestations share overlapping features and commonly co-occur in adolescents, potentially linked to shared neurobiological underpinnings (e.g., dysregulation in dopaminergic or serotonergic pathways; Jiang et al., 2019). Without effective intervention, hyperactivity/impulsivity and oppositional defiance during adolescence may persist into adulthood, elevating risks for antisocial behaviors, substance abuse, and interpersonal conflicts (Retz et al., 2021). Early identification and targeted interventions—such as cognitive-behavioral therapy (CBT), parent management training (PMT), and school-based behavioral support—can mitigate these trajectories by addressing core deficits in emotional regulation and impulse control. By fostering adaptive coping strategies and reinforcing prosocial behaviors, such approaches not only reduce immediate behavioral challenges but also promote long-term psychological resilience and social integration.

In adolescents, hostility often manifests as generalized anger and resentment toward others (Rubio-Garay et al., 2016). Hostility shares conceptual overlap with oppositional defiance, as both traits are linked to heightened self-awareness, pursuit of autonomy, and skepticism toward authority—factors that may collectively impair social and academic functioning (Zhao and Zhang, 2015). When adolescents encounter frustration, stress, or perceived injustice, they are prone to intense negative emotions like anger and resentment. If unregulated, these emotions may escalate into defiance and hostile behaviors as maladaptive coping mechanisms. Compounding this risk, adverse family dynamics (e.g., harsh parenting, inconsistent discipline) and socioenvironmental stressors (e.g., peer rejection, academic pressure) further exacerbate such behaviors (Lin et al., 2022; Wan and Tao, 2024). Effective intervention requires fostering supportive environments characterized by empathy, clear boundaries, and reinforcement of prosocial behaviors, alongside skill-building in emotional regulation and conflict resolution.

Paranoia and interpersonal sensitivity are also prevalent among adolescents. Even in the absence of objective threats, adolescents with paranoia may exhibit unwarranted distrust, hypervigilance, or defensive postures (Bird et al., 2022). Concurrently, heightened self-consciousness—coupled with tendencies toward low self-esteem and social insecurity—predisposes adolescents to overinterpret others’ intentions, excessively fear judgment, and avoid social interactions (Nie et al., 2007). Addressing these challenges necessitates interventions that promote self-reflective practices (e.g., journaling, mindfulness) and cognitive restructuring to enhance self-awareness, challenge distorted beliefs, and build resilience against perceived social threats.

The centrality indices from the undirected network analysis highlight anxiety as the node with the highest strength centrality, reflecting its pervasive influence and interconnectedness within adolescent mental health challenges. This underscores the critical need for interventions targeting anxiety to improve overall psychological well-being. Paranoia, meanwhile, exhibited the highest values in both closeness centrality and betweenness centrality, signifying its critical mediating role as a bridge linking diverse psychological issues. Paranoia predisposes adolescents to misinterpret and distrust others’ intentions, which in turn exacerbates anxiety, depression, and other comorbid symptoms while undermining treatment efficacy. These findings emphasize that reducing paranoid tendencies—through strategies like cognitive-behavioral restructuring to challenge distorted beliefs and promote healthier cognitive patterns—could disrupt pathways to secondary psychological problems. By fostering accurate social perception and reducing unwarranted suspicion, interventions may alleviate the severity of interconnected symptoms and enhance therapeutic outcomes. Thus, addressing paranoia not only mitigates its direct effects but also curtails its cascading impacts on broader mental health.

In the directed network model, the relationship between adolescent mental health and attention deficits manifests through multilayered pathways of influence, spanning from core nodes such as interpersonal sensitivity, anxiety, and paranoia to behavioral outcomes like attention deficits. This hierarchical structure reveals the dynamic complexity of psychological symptom interactions.

At the top layer, interpersonal sensitivity directly connects to multiple nodes, including anxiety, paranoia, emotional instability, hostility, sense of academic pressure, compulsive symptoms, depression, poor adaptation, psychological imbalance, and oppositional defiance, underscoring its pivotal role as a network hub. Anxiety, positioned as a mediator node, is influenced by interpersonal sensitivity and propagates effects to downstream nodes such as hostility, sense of academic pressure, emotional instability, and compulsive symptoms, highlighting its role in bridging upstream vulnerabilities and behavioral manifestations. Paranoia, another mediator node, directly impacts depression, poor adaptation, and hostility, suggesting that paranoid ideations may exacerbate depressive moods, maladaptive behaviors, and antagonistic attitudes, thereby amplifying these psychological symptoms. Collectively, these pathways illustrate how core cognitive and emotional traits (e.g., interpersonal sensitivity, paranoia) cascade through intermediate mechanisms to shape behavioral outcomes, emphasizing the need for interventions that address both upstream vulnerabilities and downstream manifestations.

The intermediate layer of the network, comprising depression, hostility, emotional instability, sense of academic pressure, poor adaptation, and compulsive symptoms, serves as a nexus of mediating pathways in the psychopathological network. Depression, influenced by paranoia, exerts downstream effects through direct connections to poor adaptation and attention deficits, highlighting its role in translating cognitive distortions (e.g., paranoid ideation) into behavioral and emotional dysregulation. Hostility, shaped by anxiety and paranoia, further propagates impacts to poor adaptation and attention deficits, amplifying functional impairments across multiple domains of mental health. Emotional instability, strongly associated with anxiety, hostility, and depression, emerges as a shared risk factor that potentiates the severity of these symptoms. Meanwhile, sense of academic pressure, rooted in interpersonal sensitivity, directly links to attention deficits and poor adaptation, underscoring its role in mediating the transition from social-perceptual vulnerabilities to behavioral challenges. Poor adaptation acts as a critical bridge, connecting to attention deficits and psychological imbalance, suggesting that maladaptive coping strategies may exacerbate attentional difficulties and emotional instability. Finally, compulsive symptoms directly influence attention deficits and oppositional defiance, indicating that compulsive behaviors may both reflect and reinforce attentional and behavioral dysregulation. Collectively, these intermediate nodes illustrate the multidirectional interplay between cognitive-emotional traits and behavioral outcomes, emphasizing the need for interventions that simultaneously address mediating mechanisms and downstream manifestations.

At the base of the network lie the terminal nodes: attention deficit, oppositional defiance, psychological imbalance, and hyperactivity/impulsivity. These nodes integrate influences from multiple upstream pathways, serving as convergent endpoints for diverse psychological and behavioral symptoms. Attention deficit directly connects to oppositional defiance and hyperactivity/impulsivity, suggesting that attentional difficulties may exacerbate defiant behaviors and impulsive actions. Oppositional defiance, in turn, links to psychological imbalance and hyperactivity/impulsivity, implying that resistant attitudes toward rules and societal expectations may amplify emotional dysregulation and restlessness. This association may stem from the inherent conflict between defiant individuals and external demands, driving heightened psychological distress and maladaptive behaviors. Psychological imbalance exhibits a direct pathway to hyperactivity/impulsivity, indicating that emotional and cognitive instability may precipitate disorganized or impulsive conduct. Such behaviors could reflect attempts to self-regulate overwhelming emotions or cope with unmet needs. Finally, hyperactivity/impulsivity, positioned as the lowest-tier node, emerges as a common behavioral endpoint influenced by multiple upstream factors, underscoring its role as a hallmark of compounded psychopathological processes. Collectively, these terminal nodes illustrate how upstream cognitive-emotional vulnerabilities coalesce into observable behavioral outcomes, emphasizing the need for interventions that target both root causes and symptomatic behaviors.

The Bayesian network reveals intricate causal pathways linking adolescent mental health issues and attention deficits. Interpersonal sensitivity, as a core node, exerts direct effects on intermediate nodes such as anxiety and paranoia, which in propagate influences to terminal behavioral nodes like attention deficit, oppositional defiance, psychological imbalance, and hyperactivity/impulsivity. Anxiety indirectly impacts psychological imbalance and attention deficits through pathways mediated by hostility and emotional instability, while paranoia contributes to these outcomes via hostility and poor adaptation. Emotional instability, functioning as a shared risk factor, demonstrates strong associations with anxiety, hostility, and depression, reflecting its multifaceted role in both precipitating and exacerbating psychological symptoms. Meanwhile, sense of academic pressure acts as a mediator bridging interpersonal sensitivity to attention deficits and poor adaptation, highlighting its critical role in translating social-perceptual vulnerabilities into behavioral challenges. Poor adaptation and compulsive symptoms further modulate attention deficits and psychological imbalance through distinct pathways. For instance, anxiety-driven increases in hostility may aggravate psychological imbalance, which subsequently undermines attention regulation. Similarly, paranoia-induced maladaptive behaviors (e.g., poor adaptation) may intensify psychological distress and attentional difficulties. These cascading effects illustrate how singular psychological issues can trigger interrelated symptoms, forming a self-reinforcing network that detrimentally impacts adolescents’ attentional functioning and overall mental health. By delineating these hierarchical and reciprocal pathways, the model underscores the necessity of multi-target interventions that address both proximal mediators (e.g., hostility, emotional instability) and distal outcomes (e.g., attention deficits), thereby disrupting the cyclical nature of psychopathological comorbidity.

Therefore, this study revealed the complex causal relationships between adolescent mental health problems and attention deficits through Bayesian network analysis. Upstream nodes—Interpersonal sensitivity, Anxiety, and Paranoia—exert their influence by directly impacting midstream nodes (Hostility, Emotional instability, Sense of academic pressure, Poor adaptation, Compulsive symptoms, etc.), which in turn act upon downstream nodes (Attention deficit, Oppositional defiant, Psychological imbalance, Hyperactivity/impulsivity). This hierarchical interaction pattern underscores that mental health problems constitute a multifactorial network. Future intervention strategies should focus on ameliorating these core nodes. Targeting Interpersonal sensitivity could involve enhancing adolescents’ social competence through social skills training. For Anxiety and Paranoia, relevant cognitive behavioral therapies offer effective intervention pathways. Concurrently, alleviating Sense of academic pressure, improving Poor adaptation, and addressing Compulsive symptoms may indirectly mitigate attention deficits. Bridging centrality analysis further provides evidence for prioritizing intervention on downstream nodes: Attention deficit (bridging strength = 0.573) and Oppositional defiant (strength = 0.5) function as critical hubs (see Figure 5), bridging the mental health and attention problem dimensions. Within the hierarchical model, they serve as pivotal transducers of behavioral manifestations. This finding demonstrates high consistency with the Bayesian network results—for instance, the relatively high bridging strength of Hostility (0.398) explains its role in the midstream, simultaneously connecting Emotional instability upstream and Oppositional defiant downstream, confirming the mechanism by which emotion dysregulation impacts behavioral control (Miles et al., 2015). Conversely, the negative BEI value (−0.02) and wide confidence interval (0.389, 0.445) for Depression suggest that interventions for adolescents with high depression require a differential approach: prioritizing the disruption of the self-reinforcing cycle of internalizing symptoms rather than directly targeting attention problems (Li and Tang, 2024). Taking all these hierarchical and cross-node interactions into account, developing comprehensive intervention programs will be crucial for simultaneously improving adolescents’ mental health status and attention levels.

This study elucidates the complex causal relationships between adolescent mental health issues and attention deficits through Bayesian network analysis. Core upper-layer nodes—interpersonal sensitivity, anxiety, and paranoia—exert direct effects on intermediate nodes (e.g., hostility, emotional instability, sense of academic pressure, poor adaptation, compulsive symptoms), which cascade to influence terminal behavioral outcomes such as attention deficits, oppositional defiance, psychological imbalance, and hyperactivity/impulsivity. This hierarchical interplay underscores the multifactorial nature of mental health challenges, where symptoms dynamically interact within a networked system. Future intervention strategies should prioritize addressing these core nodes. For instance, enhancing social competence through social skills training could mitigate interpersonal sensitivity, while cognitive-behavioral therapy (CBT) may effectively reduce anxiety and paranoia. Concurrently, alleviating academic pressure, improving adaptive functioning, and addressing compulsive behaviors may indirectly ameliorate attention deficits. A holistic approach that integrates interventions across hierarchical levels—targeting upstream vulnerabilities, mediating mechanisms, and downstream behaviors—holds promise for enhancing adolescent mental health outcomes.

From a holistic perspective, the 10 dimensions of the MSSMHS predominantly occupy upper positions in the DAG model, while the three dimensions of the SNAP-IV-26 also cluster in higher hierarchical layers. Based on their positional relationships in the network, we posit that complex interactions and causal associations exist between adolescent mental health conditions and attention deficits. While mental health issues may exacerbate attention deficits to some extent, considering biological underpinnings, mental health status is more likely to function as a primary driver of attention deficits—a directional causality empirically validated in our study. Future research should further explore the specific mechanisms of interaction between mental health and attention deficits, as well as the distinct impacts of different psychological traits on attentional functioning, to strengthen evidence for clinical interventions. Consequently, interventions for adolescents with attention deficits should not only focus on alleviating core symptoms but also prioritize mental health adjustments. Comprehensive strategies integrating psychotherapy, behavioral interventions, and family support are essential to holistically improve academic performance, social functioning, and emotional well-being.

This study explored the relationship between adolescent mental health and attention deficits using both undirected and directed network analyses. However, several limitations should be acknowledged. First, the reliance on cross-sectional data restricts causal inference, as it captures variable associations at a single time point rather than dynamic changes over time. Incorporating longitudinal designs in future research could elucidate temporal causal pathways and developmental trajectories. Second, the use of the MSSMHS and SNAP-IV-26 as primary assessment tools, while validated, may overlook multidimensional aspects of mental health and attention. Future studies could integrate multimodal measures, such as behavioral observation scales, physiological markers (e.g., heart rate variability, cortisol levels), and advanced neuroimaging techniques (e.g., fMRI, EEG), to provide a holistic evaluation across behavioral, biological, and neural levels. Third, methodological advancements are warranted. Applying dynamic network models to track symptom interactions over time or leveraging machine learning algorithms to identify high-risk subgroups could deepen our understanding of these complex relationships. Lastly, the generalizability of findings may be limited by the regional and cultural homogeneity of the sample. Expanding recruitment to include diverse geographic, socioeconomic, and cultural populations would enhance the external validity of results and support broader clinical and policy applications. Addressing these limitations will strengthen the empirical foundation for adolescent mental health research and inform the development of targeted, evidence-based interventions.

## 5 Conclusion

This study demonstrates that adolescent mental health influences attention deficits through multiple distinct causal pathways: ① interpersonal sensitivity, anxiety, emotional instability, and hostility lead to inattention; ② anxiety, paranoia, depression, and hostility contribute to hyperactivity/impulsivity; ③ interpersonal sensitivity, hostility, depression, emotional instability, and sense of academic pressure result in oppositional defiant behaviors. These findings identify precise intervention targets for distinct dimensions of attention deficits in adolescents and provide mechanistic empirical evidence for understanding the multidimensional causal architecture through which psychological symptoms impact behavioral outcomes.

## Data Availability

The original contributions presented in the study are included in the article/supplementary material, further inquiries can be directed to the corresponding authors.
